# Preventing Brain Injury in the Preterm Infant—Current Controversies and Potential Therapies

**DOI:** 10.3390/ijms22041671

**Published:** 2021-02-07

**Authors:** Nathanael Yates, Alistair J. Gunn, Laura Bennet, Simerdeep K. Dhillon, Joanne O. Davidson

**Affiliations:** 1 The Queensland Brain Institute, University of Queensland, St Lucia, QLD 4072, Australia; n.yates@uq.edu.au; 2 School of Human Sciences, University of Western Australia, Crawley, WA 6009, Australia; 3 The Department of Physiology, University of Auckland, Auckland 1023, New Zealand; aj.gunn@auckland.ac.nz (A.J.G.); l.bennet@auckland.ac.nz (L.B.); s.dhillon@auckland.ac.nz (S.K.D.)

**Keywords:** preterm asphyxia, neuroprotection, hypothermia, corticosteroids, erythropoietin, stem cells, anticonvulsants

## Abstract

Preterm birth is associated with a high risk of morbidity and mortality including brain damage and cerebral palsy. The development of brain injury in the preterm infant may be influenced by many factors including perinatal asphyxia, infection/inflammation, chronic hypoxia and exposure to treatments such as mechanical ventilation and corticosteroids. There are currently very limited treatment options available. In clinical trials, magnesium sulfate has been associated with a small, significant reduction in the risk of cerebral palsy and gross motor dysfunction in early childhood but no effect on the combined outcome of death or disability, and longer-term follow up to date has not shown improved neurological outcomes in school-age children. Recombinant erythropoietin has shown neuroprotective potential in preclinical studies but two large randomized trials, in extremely preterm infants, of treatment started within 24 or 48 h of birth showed no effect on the risk of severe neurodevelopmental impairment or death at 2 years of age. Preclinical studies have highlighted a number of promising neuroprotective treatments, such as therapeutic hypothermia, melatonin, human amnion epithelial cells, umbilical cord blood and vitamin D supplementation, which may be useful at reducing brain damage in preterm infants. Moreover, refinements of clinical care of preterm infants have the potential to influence later neurological outcomes, including the administration of antenatal and postnatal corticosteroids and more accurate identification and targeted treatment of seizures.

## 1. Introduction to Preterm Brain Injury

**Figure 1:**
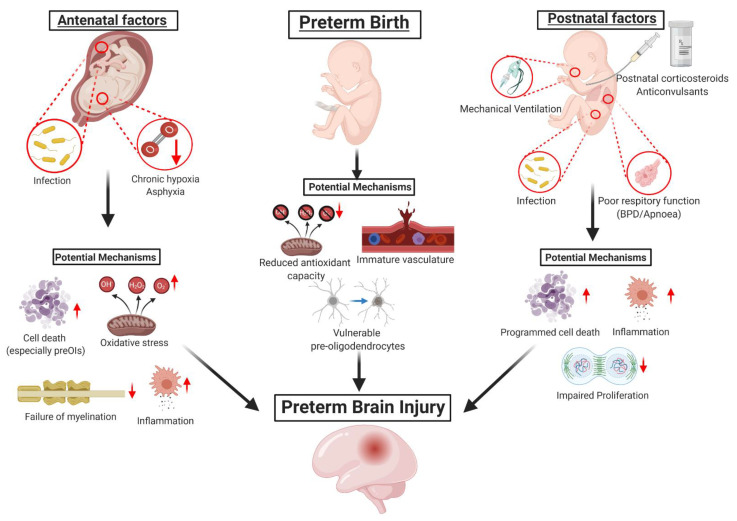
Potential factors contributing to preterm brain injury.

Premature birth, defined as birth before 37 weeks completed gestation, represents 11.1% of all live births worldwide. The rate of premature birth increased in almost all countries from 1990 to 2010 [1]. Although the mortality after preterm birth has fallen steadily over time, preterm infants continue to have very high rates of neurodevelopmental disability, including severe motor disorders such as cerebral palsy. The etiology of the disability is only partially understood, but is widely considered to be multifactorial, as illustrated in Figure 1. One of the most prevalent risk factors for perinatal brain injury is preterm birth itself, which may result from or interact with perinatal environmental and genetic factors. Disruption of nutrient or oxygen supply can arise from maternal (e.g., nutrient deficiencies, anemia), maternal–fetal (e.g., placental development) or fetal (e.g., abnormal vasculature, metabolic disorders) causes.

### 1.1. Brain Injury Associated with Hypoxia-Ischemia

Hypoxia-ischemia (HI) before, during or shortly after birth can contribute to brain injury in at least some preterm infants [2,3,4]. Older studies estimated that the prevalence of hypoxic–ischemic encephalopathy (HIE) in preterm infants was approximately 73/1000 live births, of whom ~50% were moderate to severe compared to approximately 2/1000 cases of moderate to severe encephalopathy in live term births [5]. More recently, small, retrospective studies have suggested lower rates of 1.4/1000 [6], 5/1000 [7] and 9/1000 live births [8]. Nevertheless, a recent large cohort of 115,502 preterm infants delivered in the USA between 2008 and 2011 reported that moderate to severe HIE occurred at a rate of 37.3/1000 babies born before 37 weeks of gestation [9]. Further, this study demonstrated an inverse relationship between lower gestational age at birth and greater mortality and morbidity, such that infants born before 28 weeks gestational age had an overall rate of 120/1000 live births. These studies suggest that acute asphyxia around the time of birth is an important contributor to brain damage in preterm infants.

Some of the controversy surrounding the prevalence of asphyxia in preterm infants may stem from the fact that identifying that events have occurred is more challenging than in infants over 36 weeks gestational age, as reviewed in [2]. For example, healthy preterm infants have lower body tone than term infants, making it more difficult to identify hypotonia. Nevertheless, at least in late preterm infants from 32 weeks gestation onward, it may be feasible to appropriately adapt the clinical neurological criteria used to diagnose HIE in near-term infants. Further, the impacts of HIE on childhood outcomes, particularly in infants born extremely preterm, are difficult to disentangle from the effects of preterm birth itself [2].

In addition to acute HI as discussed above, many preterm infants are exposed to chronic hypoxia before birth, as shown by intrauterine growth restriction (IUGR)/small for gestational age (SGA), defined as birthweight below the 10^th^ percentile [10]. IUGR/SGA is multifactorial. In the developed world, most cases are related to placental insufficiency but malnutrition and chromosomal abnormalities are also potential causes. IUGR/SGA is associated with increased risk of death, developmental impairment, cerebral palsy and cardio-metabolic disease [10,11,12]. The adverse outcomes associated with IUGR/SGA are at least in part attributed to increased vulnerability to HI at birth [13], likely relating to reduced cardiac glycogen storage and therefore less ability to adapt to repeated severe hypoxia in labor [14].

Preterm infants may also experience intermittent HI after birth, due to conditions such as apnea of prematurity causing repeated periods of mild hypoxia, which are associated with neurodevelopmental and motor impairment [15]. Further, preterm infants may develop bronchopulmonary dysplasia (BPD), a chronic respiratory disease that is associated with frequent periods of hypoxia and neurodevelopmental impairment, particularly if it is moderate or severe [16].

### 1.2. Brain Injury Associated with Infection and/or Inflammation

Chorioamnionitis (infection of the fetal membranes) is associated with 11–40% of all preterm births [17]. The incidence of chorioamnionitis increases substantially with decreasing gestational age, such that it is associated with only ~4% of term deliveries but 94% of deliveries at 21–24 weeks of gestation [18]. Chorioamnionitis is characterized by invasion of microorganisms including bacteria, viruses and fungal species into the amniotic cavity, by many routes, including ascending from the lower genital tract, from the placenta, accidental introduction via invasive procedures such as amniocentesis, contamination via intrauterine contraceptive devices or retrograde spread via the fallopian tubes [19]. Chorioamnionitis is an independent risk factor for adverse brain development, including intraventricular hemorrhage (IVH), neurological impairment and cerebral palsy [20,21,22,23].

During exposure to intrauterine infection/inflammation, the fetal immune system responds by releasing pro-inflammatory cytokines that induce a fetal inflammatory response syndrome (FIRS) [24]. Fetal systemic inflammation is associated with induction of cerebral inflammation and adverse neurodevelopmental outcomes [25].

### 1.3. Vulnerabilities of the Preterm Brain to Injury

Finally, and not least, the preterm infant is understandably not fully adapted to the ex-utero environment, because of reduced access to suitable nutrition, high oxygen exposure, large blood pressure fluctuations related to fluid shifts, immune system challenges, and exposure to a highly pro-inflammatory environment.

Moreover, there are some unique characteristics that contribute to the vulnerability of the preterm brain to injury. Firstly, cell division and maturation are in progress. There is considerable evidence suggesting that pre-oligodendrocytes are particularly susceptible to injury and death [26]. Oligodendrocytes develop according to a well-characterized lineage, with pre-myelinating oligodendrocyte progenitors developing into pre-oligodendrocytes, which then develop into the myelinating immature and mature oligodendrocytes, as reviewed in [27]. The developmental window associated with the highest risk of periventricular white matter injury (23–32 weeks postconceptional age) coincides with a high proportion of pre-oligodendrocytes and this risk declines with the onset of differentiation of the pre-oligodendrocytes into the myelinating immature oligodendrocytes [28,29,30]. In 2-day-old rat pups, when pre-oligodendrocytes are the dominant lineage, are more susceptible to hypoxic–ischemic injury of white matter than 7-day-old rat pups, when immature oligodendrocytes are predominant [29]. Further, in 0.65 gestation fetal sheep, there was greater oligodendrocyte cell death in the medial periventricular white matter, in which pre-oligodendrocytes are the predominant lineage, compared to the lateral periventricular white matter, which has a higher proportion of immature oligodendrocytes [31]. 

The relative vulnerability of the pre-oligodendrocyte to HI compared to the immature oligodendrocyte may, in part, be mediated by greater susceptibility to oxidative stress, as shown in vitro by greater death of pre-oligodendrocytes in response to oxidative stress induced by depletion of intracellular glutathione [32]. Pregnancy itself is associated with increased oxygen demand and rate of production of reactive oxygen species leading to elevated oxidative stress and lipid peroxidation compared with non-pregnant women, as reviewed in [33]. Further, there is an increase in reactive oxygen species generated by the placenta of women experiencing pre-eclampsia. Compared to adults, the newborn infant has low levels of endogenous antioxidant capacity, including lower levels of plasma antioxidants such as vitamin D, beta carotene and sulfhydryl groups and lower levels of plasma metal binding proteins and reduced activity of erythrocyte superoxide dismutase, increasing its vulnerability to elevated oxidative stress, such as that which occurs in response after ischemia-reperfusion [33,34].

Next, the preterm brain has a comparatively underdeveloped vasculature, which matures relatively late in gestation; these increases in vascular density and cross-sectional area continue well into adulthood [35,36]. The most vulnerable brain region is the germinal matrix due to the higher vascular density, greater immaturity, and reduced structural integrity of blood vessels compared to both white and grey matter, at 16 to 32 weeks of gestation. Germinal matrix-IVH (GM-IVH) is a critical complication of prematurity involving hemorrhage that starts at the germinal matrix and progresses to the lateral ventricles [37]. It is inversely associated with both gestational age and birth weight and occurs in approximately 20% of very low birthweight preterm neonates (weighing < 1000 g at birth). Approximately 30–50% of preterm infants with serious IVH will develop post-hemorrhagic ventricular dilation or hydrocephalus. Although this may spontaneously resolve in some infants, approximately 25% require insertion of a shunt to alleviate progressive hydrocephalus. Need for a shunt after severe IVH has been associated with adverse neurodevelopmental and growth outcomes at 18 to 22 months compared to children who did not require a shunt [38]. Another serious complication of GM-IVH is periventricular hemorrhagic infarction, which is associated with significant cognitive and/or motor abnormalities in two thirds of survivors [39].

## 2. Neurological Outcomes

Infants born preterm are at high risk for neurodevelopmental disorders, including cerebral palsy [40]. The risk is greatest in extremely preterm infants (<28 weeks gestation), who have highest rates of poor neurological outcomes such as cognitive impairment, hearing loss and retinopathy of prematurity [41]. 

Preterm infants examined at term equivalent age show widespread changes in white matter diffusivity, indicating compromised white matter integrity, which is often observed in neurodevelopmental disorders such as cerebral palsy [42]. Critically, extremely preterm infants at term-corrected age show reduced cerebral cortical and deep nuclear grey matter volumes, and increased cerebrospinal fluid volumes [43]. Very premature infants (infants born ≤ 32 weeks’ gestational age) show high rates of white matter (31.6%) and grey matter (21.1%) abnormalities on MRI, which are predictive of poor neurodevelopmental outcomes at 9 years of age [44]. Repeated MRI scans of very preterm infants (23 to 30 weeks GA) show that 49% exhibit changes in brain structure and regional volumes shortly after birth, which increases to 92% by near-term gestational age (GA) [45]. The nature of brain pathology evolves over time, including the appearance of hemorrhagic lesions (germinal matrix and intraventricular), changes in signal intensity (reflecting necrotic or demyelinating processes) and ventricular dilatation [45].

### The Quest to Develop Treatment Strategies to Reduce Preterm Brain Injury 

A key challenge in developing treatment strategies to reduce brain injury in preterm infants is the large number of different injurious phenomena that these infants can be exposed to before, during or after birth. As highlighted above, these include preterm birth itself, developmental vulnerability to injury at a particular gestational age, chronic hypoxia in utero, acute asphyxia around the time of birth, infection/inflammation and poor respiratory function after birth. Further, many therapies required for neonatal intensive care such as ventilation, may decrease mortality but increase risk of brain injury [46]. To date therapies for preterm infants have been largely ineffective in improving long-term neurological outcomes and there has been at best modest improvements over time [47,48,49]. However, exciting potential treatments are being investigated in preclinical or clinical trials that may help to reduce the burden of brain injury in the preterm infant.

## 3. Treatments Currently in Clinical Use for Preterm Infants

### 3.1. Antenatal Corticosteroids

Maternal glucocorticoid therapy is recommended for cases of threatened or current preterm labor to promote fetal lung development. The safety and benefits of antenatal glucocorticoids were observed in the EPIPAGE cohort study (Table 1), which showed that very preterm infants (<32 weeks) had a reduction in white matter injury (OR = 0.60, 95% CI = 0.46–0.79) and death (OR = 0.61, 95% CI = 0.41–0.91), but no changes in rates of adverse neurological outcomes at 5 years old [50]. A recent meta-analysis examining 30 trials of antenatal glucocorticoids for women at risk of giving preterm birth found a reduction in infant perinatal death, neonatal death, respiratory distress syndrome, need for mechanical ventilation, and infection therapy [51], with a reduced incidence of IVH (RR = 0.55, 95% CI 0.40 to 0.76, 16 studies, participants = 6093). The only study that reported neurodevelopmental outcomes in childhood did not show a significant effect of antenatal glucocorticoids (RR = 0.64, 9% CI 0.14 to 2.98), but was very underpowered, with only 82 participants [52]. Repeated doses, given more than 7 day apart seem to improve short-term outcomes, without increasing mortality [53]. Importantly, a recent randomized double-blind controlled trial including 1509 fetuses who received antenatal corticosteroids, showed no significant difference in neurosensory disability at 2 years of age between those treated with dexamethasone and those treated with betamethasone [54]. Consistent with this, a recent meta-analysis of 45 trials including 11,227 women and 11,878 infants, found no difference between dexamethasone and betamethasone on neonatal death, neurodevelopmental disability, IVH and birthweight [55]. In contrast to the consistent short-term benefits seen in clinical trials, preclinical studies from a wide range of animal models have raised concerns that antenatal steroids may have adverse effects on neurological outcomes. For example, weekly maternal betamethasone starting as early as 0.63 gestation in sheep reduced myelination in multiple brain regions [56]. In the context of the more rapid developmental of the fetal sheep compared to humans, this represents a relatively more prolonged exposure than it would be for a human. Interestingly, in preterm fetal sheep, exposure to a clinically relevant dose of maternal dexamethasone was associated with transient evolving epileptiform activity, consistent with electrographic and clinical seizures [57]. However, no neural injury or microglial activation was seen at postmortem and maturation of the EEG was enhanced, suggesting there were no adverse outcomes [57]. Reassuringly, clinical MRI studies have demonstrated that antenatal betamethasone or hydrocortisone did not influence regional brain volumes in humans [58,59].

Of greater potential concern, studies in preterm fetal sheep suggest potential deleterious effects after acute HI. For example, maternal dexamethasone given 15 min after fetal asphyxia was associated more severe neuronal loss in the hippocampus and basal ganglia and greater loss of both total and immature/mature oligodendrocytes in the periventricular white matter [60]. In the same experimental paradigm, maternal dexamethasone treatment was associated with a significantly greater fall in carotid artery blood flow and cerebral oxygenation measured using near infrared spectroscopy, in the first 6 h after asphyxia, despite similar brain activity, suggesting that greater hypoperfusion and impaired cerebral oxygenation during the latent phase may underlie the exacerbation of neural injury [61]. 

When a single i.m. injection of maternal dexamethasone was given 4 h before asphyxia, induced by 25 min of complete umbilical cord occlusion in preterm fetal sheep, severe, cystic brain injury was seen, with increased numbers of seizures, worse recovery of brain activity and increased arterial glucose levels compared to diffuse injury after asphyxia alone [62]. Importantly, these findings could be replicated by glucose infusions before asphyxia, suggesting that dexamethasone-induced hyperglycemia may transform diffuse injury into cystic brain injury after asphyxia in the preterm fetal sheep [62]. These data suggest that there is potential for antenatal corticosteroids to have adverse effects on the small subset of preterm infants that may be exposed to asphyxia before or during birth but this has not been specifically investigated in clinical studies. However, we must bear in mind that the occurrence of acute asphyxia in a fetus after administration of antenatal steroids is both unanticipated and unpreventable, while the short-term benefits of antenatal corticosteroids in general are well established.

### 3.2. Postnatal Glucocorticoids

Preterm infants with BPD have a prolonged oxygen requirement after birth, with greatest risk of BPD with decreasing gestational age. Approximately 50% of extremely preterm infants (<28 weeks) and up to 80% of infants born <25 weeks develop BPD [76,77]. Given the strong inflammation associated with BPD, the use of anti-inflammatory agents such as postnatal glucocorticoids was initially widely embraced as a treatment strategy. However, evidence of an association between high-dose postnatal glucocorticoid use and the development of cerebral palsy led to a marked decline in their use in recent decades [78]. Postnatal dexamethasone treatment was associated with negative neurodevelopmental consequences including increased risk of developing CP (OR = 4.45) and developmental delay (OR = 2.87) [45]. Furthermore, high-dose postnatal dexamethasone reduces total intracranial, cerebral tissue, and cortical grey matter volume (Table 1) [63,64]. 

More recent studies suggest that key factors affecting the relative benefits and risks of postnatal glucocorticoid therapy, include dosing and timing of treatment as well as the underlying relative risk of developing cerebral palsy. High-dose dexamethasone exposure decreased brain weight, delayed neurological development [79], reduced skilled motor coordination and altered posture [80], reduced cerebellar size [81], reduced hippocampal cell proliferation [82], and induced apoptosis in the hippocampus and striatum in rodents [83]. Moreover, high-dose dexamethasone in preterm infants is associated with a trend for increased PVL [84,85]. Although high-dose glucocorticoids are associated with poor neurodevelopment, contemporary low-dose dexamethasone protocols (approximately 10% of the doses used in the 1990s) appear to improve infant ventilation outcomes without significant adverse neurodevelopmental outcomes [86,87]. Recently, a study in moderately preterm lambs (0.86 gestation, full-term brain equivalent) found no obvious risk of neurological harm with high- or low-dose tapered postnatal dexamethasone over 7 days for a cumulative dose 0.27 and 2.67 mg/kg, respectively [88]. There were equivocal outcomes for brain lesions, frontal cortex volumes (white and grey matter), frontal cortex thickness, hippocampus volumes, and gross morphometric measurements [88]. 

Recent systematic reviews of the relative benefits and risks of both postnatal early (<8 days) or late (>7 days) glucocorticoid therapy concluded that the benefits may not outweigh the risks of treatment [65,66]. Systemic corticosteroids administered < 8 days after birth were associated with beneficial short-term effects for respiratory outcomes but a worrying long-term increase in the risk of cerebral palsy [65]. When systemic corticosteroids were administered > 7 days after birth, similar short-term benefits were seen, without a significant increased risk of cerebral palsy [66]. A meta-analysis by Onland et al. [67] found that with moderately-early (7–14 days after birth) dexamethasone therapy, higher cumulative doses result in reduced rates of BPD without increasing the risk of neurodevelopmental impairment. Further, the risk of mortality and cerebral palsy decreased with each incremental mg/kg cumulative dexamethasone dose. However, this may be explained by the benefit of postnatal dexamethasone interacting with the risk of chronic lung disease and BPD, given that prolonged mechanical ventilation is an independent risk factor for cerebral palsy [89]. The most important finding of this research is that treating preterm infants at a low risk for BPD increases risk of cerebral palsy, whereas treating high-risk infants for BPD lowers risk of cerebral palsy [90,91]. 

### 3.3. Magnesium Sulfate (MgSO_4_)

Magnesium is an endogenous anti-excitotoxic agent that acts by binding to the magnesium site on the NMDA receptor, inhibiting these glutamatergic channels [92]. This has led to interest in the use of magnesium sulfate as neuroprotective treatment for both term and preterm babies. However, the data remain controversial. 

Experimentally, some studies in neonatal rodents have suggested neuroprotection with MgSO_4_ before or after HI. However, it is critical to appreciate that magnesium is a potent vasodilator and so can increase heat loss, leading to iatrogenic hypothermia in small animals. Systematic review of the literature suggests no benefit in rodents, piglets and fetal sheep in studies with good temperature control [93].

Meta-analysis of large randomized controlled trials of MgSO_4_ administered to women in preterm labor found that it was associated with a small but significant reduction in the risk of cerebral palsy and gross motor dysfunction in early childhood (Table 1) [68,69]. However, there was no net effect on overall death and disability [94,95]. Further, two of the five randomized controlled trials included in the original meta-analyses have now followed children up to school age and show no significant improvement in cognitive, behavioral or functional outcomes [47,48]. Given the lack of long-term clinical benefit, there is considerable ongoing controversy around whether MgSO_4_ has true neuroprotective effects in the preterm infant. 

### 3.4. Anticonvulsants

The most common age for seizures is <1 year old [96], and is higher still in the neonatal period (<28 days, [97]). Seizures in preterm neonates are particularly common (clinical seizures term vs. preterm incidence 2.0 vs. 11.1 per 1000 live births [98]). Single seizures are unusual in neonates, and recurrent seizures are common if left untreated [99]. Neonatal seizures are associated with poor neurodevelopment and preterm infants with seizures have been reported to have worse outcomes than term infants with seizures [100]. The direction of causality though remains highly unclear.

A recent survey of 193 neonatal physicians indicated that the barbiturates phenobarbital and phenytoin are the most common anticonvulsant medications used to control neonatal seizures [101]. However, the use of these GABAergic drugs is controversial due to potential adverse effects on short- and long-term brain development, and the lack of robust evidence for efficacy [49]. Systematic reviews of barbiturate use show that less than 50% of neonates respond to first line therapy, and none when they are used as a second-line therapy [102]. Indeed, clinical signs of improvements may be due to the sedative effects of these drugs, rather than reducing seizure activity and underlying brain dysfunction [49]. The evidence for a beneficial effect of anticonvulsant drugs in neonates is so limited that a 2007 Cochrane review concluded that “anticonvulsant therapy to term infants in the immediate period following perinatal asphyxia cannot be recommended for routine clinical practice, other than in the treatment of prolonged or frequent clinical seizures” [103].

Preclinical evidence in the developing rat brain suggests that many anticonvulsant drugs are also neurotoxic at clinical and subclinical doses [104]. Phenobarbital treatment was associated with reduced proliferation in the dentate gyrus and reduced expression of neuronal markers and neuronal transcription factors and neurotrophins in P4–P6 neonatal rats [105]. In P7 rats, clinically relevant doses of phenytoin, phenobarbital, diazepam, clonazepam, vigabatrin or valproate were associated with widespread apoptotic degeneration, reduced expression of pro-survival neurotrophins, and a significant reduction in brain weight after eight days [104]. Further, clinical doses of phenobarbitone, phenytoin and lamotrigine in P7 neonatal rats were associated with impaired striatal synaptic development between P10 and P18 [106]. Encouragingly, levetiracetam treatment did not impair synaptic development [106], consistent with a previous study showing that it did not induce cell death in the P7 rat brain [107]. 

The potential harm and lack of efficacy of GABAergic agonists may be due to developmental changes in GABA receptor responses. Early in development, GABA receptors may respond to stimulation with excitation, rather than the inhibition observed in adults [108] due to high intracellular Cl- concentration resulting from high Na-K-2Cl (NKCC1) expression, which mediates Cl- entry and low K-Cl cotransporter KCC2 expression, which mediates Cl- exit from cells [109,110]. With the upregulation of the potassium-chloride co-transporter KCC2 during the early postnatal period, chloride ions can be extruded and GABA and glycine become inhibitory. It has been shown that late gestation HI reduced neuronal KCC2 expression, impairing hippocampal CA3 inhibitory tone in juvenile rats [111]. Impaired inhibitory tone after HI may lower the seizure threshold and contribute to the lack of efficacy of GABAergic agonists in neonates. This suggests that GABA agonists may be developmentally inappropriate in infants. This led investigators to suggest the use of more developmentally appropriate anticonvulsant treatments, such as activation of NKCC1 with the loop diuretic bumetanide in infants 33–44 weeks postmenstrual age [49,112]. However, an exploratory trial in term infants did not show evidence for seizure control and there was an apparent increased risk of hearing loss [113]. Interestingly, postnatal administration of the potential neuroprotective treatment recombinant erythropoietin (rEpo) has been shown to attenuate the loss of KCC2, associated with late gestation in utero HI brain injury in rats, potentially reversing the deficits in inhibitory circuit formation and greater susceptibility to seizure activity [114].

In summary, there are major challenges in treating preterm infants with seizures. Current first-line anticonvulsants are at best moderately effective, and potentially harmful. Improved understanding of the specific mechanisms underlying neonatal seizures and the development of appropriate anticonvulsant therapies are key research priorities.

## 4. Potential Neuroprotective Treatments that Have Been Investigated in Clinical and Preclinical Trials for Use in Preterm Infants 

### 4.1. Erythropoietin

Erythropoietin (Epo) has a central role in erythropoiesis and has been widely used to prevent postnatal anemia in premature infants [115]. In addition, rEpo has shown many potentially neuroprotective effects, including promoting expression of anti-apoptotic more than pro-apoptotic genes, inhibiting caspase activation, and attenuating inflammation and oxidative stress, as reviewed in [116]. There is substantial evidence from preclinical models that treatment with rEpo given shortly after HI is neuroprotective in the developing brain. In P7 rats, repeated injections of rEpo (5000 IU/kg on days one, two and three) reduced brain injury and was more effective than a single (5000 IU/kg) dose or three injections with 2500 or 30,000 IU/kg [117]. In preterm-equivalent fetal sheep, treatment with a prolonged infusion of rEpo (5000 IU/kg by slow push, then 832.5 IU/h from 30 min to 72 h) after severe asphyxia induced by complete umbilical cord occlusion, was partially neuroprotective, reduced seizure burden and improved the recovery of EEG power and reduced apoptosis and inflammation, three days after HI [118].

rEpo treatment has shown potential to support the restoration of neural structure and function over the long-term recovery from HI. In P7 rat pups, treatment with repeated injections of rEpo (1000 U/Kg) from 48 h after HI, increased neurogenesis and oligodendrogenesis, improved oligodendrocyte maturation, and restored myelination two weeks after HI [119]. Similarly, postnatal treatment with five injections of rEpo (2000 U/Kg) in rat pups exposed to transient HI at embryonic day 18 restored HI-induced functional deficits in gait and social interaction at P28–30 and attenuated structural abnormalities in the white matter and subcortical grey matter structures seen using diffusion tensor MRI at P35–40 [120]. 

Importantly, rEpo also appears to be beneficial in models of preterm infection/inflammation. A single injection of rEpo (5000 IU/Kg) after maternal LPS injection in rats at 18–19 days gestation was associated with reduced IL6, IL1 and TNF-α concentrations, apoptosis and demyelination at p7 [121]. In preterm fetal sheep with endotoxin-induced brain damage, intravenous rEpo (5000 IU/Kg) injections administered once daily for 3 days, reduced axonal damage, microglial and astrocytic responses in the white matter and improved myelination [122].

Clinically, phase 1 and 2 trials have provided strong evidence of the safety of rEpo treatment in low birth weight infants, prematurely born infants and full-term neonates with HIE [116,123]. In a small clinical trial in very preterm infants, rEpo (500 U/kg intravenously every other day for 2 weeks started within 72 h of birth) was associated with improved neurodevelopmental outcomes at 18 months of age (Table 1) [70]. Meta-analysis of 1133 very preterm infants (approximately 32 weeks gestation) from four randomized control trials who received prophylactic rEpo treatment suggested a reduced incidence of severely impaired neurodevelopmental scores at 18–24 months post menstrual age [123]. However, more recently, a randomized, double-blind trial of high-dose rEpo administered to extremely preterm infants from 24 h after birth until 32 weeks post menstrual age, had no effect on the risk of severe neurodevelopmental impairment or death at 2 years of age [71]. Further, a similar study of 448 infants randomized to receive repeated doses of rEpo started within 3 h of very preterm birth found no effect on neurodevelopmental outcomes at 2 and 5 years of age [72,73]. It is possible that the relatively delayed start (within 24 h) and infrequent dosing regimen in Juul et al. [71] may have contributed to the lack of neuroprotection seen in this study as infants received 1000 IU/Kg rEpo every 48 h for a total of 6 doses followed by 400 IU/Kg 3 times per week, whereas the study in preterm fetal sheep showing partial neuroprotection used early initiation of treatment at 30 min and a prolonged infusion to maintain a stable plasma rEpo concentration [118]. This highlights the importance of optimizing the dosing regimen and therapeutic window of opportunity for treatment in a large animal translational model prior to the start of a clinical trial.

One potential solution to the need for frequent dosing with rEpo could be the use of darbepoetin (Darbe), the long-acting erythropoiesis-stimulating agent, which has been shown to have a half-life that is 3-fold longer than rEpo in term neuroprotection studies [124,125]. In a small clinical study comparing rEpo, Darbe and placebo, administration of either rEpo or Darbe was associated with higher cognitive scores and a reduced incidence of cerebral palsy [126].

### 4.2. Therapeutic Hypothermia

Mild therapeutic hypothermia induced by cooling the head or body of an infant is now routine clinical care for term infants suffering HIE [127,128]. The success of therapeutic hypothermia as a treatment strategy is likely attributable to its wide array of mechanisms of action, including reducing metabolism, suppressing programmed cell death pathways, and attenuating the inflammatory response and protecting mitochondrial function, as reviewed in [129].

There is compelling clinical and experimental evidence that therapeutic hypothermia can reduce neuronal loss and improve neurological outcome in term and near-term infants with moderate to severe HIE [127,128]. Meta-analysis of 11 randomized controlled trials of either selective head cooling or whole-body cooling initiated within 6 h of birth, involving 1505 term and late preterm (35–36 weeks) infants with moderate/severe HIE found highly consistent beneficial effects after hypothermia [128]. Therapeutic hypothermia was associated with reduced mortality or major neurodevelopmental disability by 18 months of age (relative risk (RR) 0.75 (95% confidence interval (CI) 0.68 to 0.83). Long-term follow up of these studies suggests that improvements in outcome persist into middle childhood after mild induced hypothermia for HIE [130,131,132]. Although these trials included late preterm infants over the age of 35 weeks gestation, little is known about the safety and efficacy of therapeutic hypothermia for younger preterm infants. 

Preclinical evidence suggests that hypothermia is also effective at reducing brain damage in the preterm brain. For example, mild cerebral hypothermia started 90 min after asphyxia induced by complete umbilical cord occlusion and continued for 3 days was associated with a marked reduction in loss of neurons and immature oligodendrocytes, restored carotid artery blood flow and EEG frequency to sham control levels in 0.7 gestation fetal sheep [133]. Similarly, in the same model, hypothermia started 30 min after 25 min of complete umbilical cord occlusion was associated with reduced neuronal loss and microglial induction in the striatum, faster recovery of spectral edge frequency, reduced seizure burden and less suppression of EEG amplitude [134]. However, when treatment was delayed until 5 h after umbilical cord occlusion, no neuroprotection was seen suggesting that the therapeutic window of opportunity may be even narrower than in the term brain, where limited neuroprotection can be achieved with hypothermia started 5.5 h after 30 min of global cerebral ischemia [135]. 

Although it seems plausible that hypothermia will be effective at reducing brain damage in the preterm brain after an acute hypoxic–ischemic event such as perinatal asphyxia, concerns remain regarding its safety. Mild hypothermia (33−35 °C) has been associated with slowing of the atrial pacemaker and intracardiac conduction, with sinus bradycardia, decreased left ventricular contractility and cardiac output, as reviewed in [136]. A small retrospective study of 31 preterm neonates born at 34–35 weeks gestation with HIE and treated with hypothermia suggests that use of hypothermia is feasible in this age group but that caution is warranted due to a higher risk of mortality and side-effects, such as hyperglycemia, compared to term neonates treated with hypothermia (Table 1) [74]. In a retrospective uncontrolled cohort analysis of 30 preterm infants 33–35 weeks gestation with HIE who received whole-body hypothermia, there was a concerning incidence of combined outcome of death and neurodevelopmental impairment and complications, including coagulopathy, early clinical seizures, arterial hypotension, persistent metabolic acidosis and thrombocytopenia [75]. However, interpretation of these studies is limited due to their retrospective nature, small cohort size and lack of normothermia controls.

The Preemie Hypothermia for Neonatal Encephalopathy trial (NCT01793129) is currently active, investigating the use of mild therapeutic hypothermia for preterm infants between 33 and 35 weeks gestational age who present at less than 6 h postnatal age with moderate to severe neonatal encephalopathy (Clinical Trial’s Register). Infants are randomized to receive 72 h of whole-body hypothermia or normothermia.

## 5. Potential Neuroprotective Treatments Showing Promise in Preclinical Studies of Preterm Brain Injury

### 5.1. Melatonin

Melatonin (*N*-acetyl-5-methoxytryptamine) is a naturally occurring indolamine secreted by the pineal gland to regulate circadian rhythm that also has antioxidant properties [137]. Exogenous melatonin has potential to be a prophylactic treatment for fetuses at high risk of perinatal hypoxia as it readily crosses the placenta [137]. However, recent large animal studies suggest that a part of the neuroprotective effects observed with melatonin treatment were attributable to the diluent, ethanol. 

In preterm fetal sheep at 0.7 gestation, maternal low-dose melatonin infusion from 15 min before asphyxia until 6 h after, was associated with faster recovery of EEG activity, delayed onset of seizures, improved survival of mature oligodendrocytes, and reduced microglial activation in the periventricular white matter [138]. However, the ethanol vehicle was independently associated with reduced duration of fetal seizures and improved neuronal survival in the striatum, albeit with worse neuronal survival in the hippocampus and less white matter proliferation compared to saline treatment. These data suggest complex confounding effects of ethanol. Direct fetal infusion of melatonin (2.6 mg dissolved in ethanol, over 24 h) in preterm (0.7 gestation) fetal sheep, starting 2 h after HI showed region specific improvement in white matter damage 10 days after HI [139].

Further supporting neuroprotective effects of melatonin and ethanol, they have been shown to be similarly neuroprotective in the term brain after HI. In a recent study in neonatal piglets, treatment with high-dose melatonin (18 mg/kg) in conjunction with therapeutic hypothermia, significantly improved recovery of aEEG activity, improved cerebral energy metabolism seen using magnetic resonance spectroscopy and reduced TUNEL-positive cell death after asphyxia [140]. However, this study also showed that the ethanol used to improve melatonin solubility was independently associated with partial protection, including recovery of aEEG and reduced cell death, particularly of oligodendrocytes. Administration of high-dose melatonin (5 mg/kg/h over 6 h) dissolved in ethanol started immediately after HI in postnatal term piglets significantly augmented neuroprotection from therapeutic hypothermia on both magnetic resonance spectroscopy markers of anaerobic stress, and histopathology [141]. When an ethanol-free proprietary melatonin formulation using excipients considered safe for use in neonates was given to neonatal piglets at 2 and 26 h after HI combined with cooling from 2 to 26 h, localized additive protective effects were seen in the sensorimotor cortex, but not other cortical, subcortical or white matter regions examined, with melatonin showing a very narrow window of opportunity for effective treatment [142]. These studies suggest that the combination of melatonin and ethanol has the potential to effectively reduce brain damage after HI in both the preterm and term brain. The question is whether ethanol will ever be viewed as an acceptable drug to be trialed as a neuroprotective therapy for preterm infants.

Pretreatment with maternal melatonin has been shown to be beneficial in a mouse model of maternal inflammation, with reduced preterm birth and preterm brain injury seen when pregnant mothers of embryonic day 16.5 mice received melatonin before the induction of inflammation using lipopolysaccharide [143]. A limitation of this study is that melatonin was dissolved in dimethyl sulfoxide (DMSO) but no vehicle control group was included to establish whether the vehicle may be contributing to the observed effect given that DMSO has long been known to have neuroprotective effects [144,145].

Melatonin given in combination with erythropoietin prevented post-hemorrhagic hydrocephalus of prematurity in rat pups [120]. In this study, prenatal chorioamnionitis was induced at embryonic day 18 by transient uterine artery occlusion for 60 min followed by intra-amniotic injection of lipopolysaccharide. On postnatal day one, IVH was induced by injection of lysed red blood cells into the lateral ventricles. Pups received 6 doses of rEpo on P2–P5, P7 and P9 and 9 doses of melatonin (20 mg/kg i.p.) on day P2–P10. Combined rEpo and melatonin treatment prevented the development of multiple hallmarks of post-hemorrhagic hydrocephalus of prematurity, including macrocephaly and neurodevelopmental delay and reduced ventriculomegaly. However, rEpo and melatonin were not given as separate treatments in this study, so the relative contribution of melatonin cannot be determined.

Maternal pre-treatment with melatonin has been shown to reduce preterm birth and perinatal brain injury in a mouse model of maternal inflammation induced by lipopolysaccharide [143]. Melatonin pre-treatment was associated with lower pro-inflammatory cytokines in the uterus and placenta as well as a significant reduction in LPS-induced fetal neuro-inflammation.

Small clinical studies suggest that melatonin is not associated with adverse outcomes and may improve survival of neonates with septic shock and reduce lung injury associated with ventilation in preterm infants [146,147]. A small randomized trial in term neonates with HIE reported that five enteral doses of melatonin (10 mg/kg) given in combination with therapeutic hypothermia, reduced seizures and white matter abnormalities on MRI at two weeks of age, and improved survival without neurological abnormalities at six months of age [148]. Long-term follow up has not been reported. 

### 5.2. Vitamin D Supplementation

Maternal vitamin D deficiency appears to be a significant risk factor for preterm birth [149,150], primary cesarean section (a cesarean section performed on a woman for the first time) [151], small for gestational age, and pre-eclampsia [152,153,154], such that increasingly premature infants are more likely to be vitamin D deficient at birth [155,156]. Meta-analysis has demonstrated an association between preterm birth risk and maternal vitamin D deficiency [153]. Further, a recent study has shown an association between umbilical cord vitamin D levels and adverse neonatal outcomes, including increased risk of preterm birth, neonatal respiratory distress syndrome and increased risk of hospitalization in the first year of life [157]. There is also an association between a vitamin D receptor polymorphism and spontaneous preterm birth [158]. 

Vitamin D plays several important roles during development. Vitamin D has been shown to influence normal fetal brain development, including cell differentiation, neurotrophic factor expression, cytokine regulation, neurotransmitter synthesis, intracellular calcium signaling, antioxidant activity and expression of genes and proteins involved in neuronal differentiation, structure and metabolism [159]. Maternal deficiency in rodents has been implicated in impaired placental function, increased maternal and fetal glucocorticoid exposure, and changes in behavior [160,161,162]. Epidemiological data suggests that maternal vitamin D deficiency may be associated with adult schizophrenia [163], poor child language development [164,165], and a growing body of evidence for autism-like traits [162,166,167]. Nevertheless, the direction of causality is unproven.

Vitamin D supplementation has shown a range of neuroprotective effects in preclinical models of brain injury, including immunomodulatory and anti-inflammatory effects. Indeed, vitamin D has been shown to be a key immunomodulator and regulator of pro-inflammatory Th 17 lymphocytes in adult stroke patients [168,169]. In adult male mice, administration of the active form of vitamin D, 1,25-dihydroxyvitamin D3 (1,25-VitD3) for 5 days prior to stroke induced by middle cerebral artery occlusion for one hour followed by 23 h of reperfusion, was associated with a significant reduction in infarct volume and reduced expression of the pro-inflammatory cytokines interleukin (IL)-6, Il-1β, IL-23a, TGF-β and NADPH oxidase-2 [170]. Interestingly, 1,25-VitD3 was shown to prevent autism-related phenotypes in the adult offspring in a mouse model of maternal immune activation but this was not associated with a reduction in pro-inflammatory cytokines in either the dam or the fetal brain, suggesting an alternative mechanism of action [166]. In a mouse brain endothelial cell culture model designed to mimic the blood-brain barrier in vitro, administration of 1,25-VitD3, prevented the decrease in barrier function as measured by transendothelial electrical resistance, permeability of FITC-dextran, decrease of tight junction proteins, activation of NF-k β, and increase in matrix metalloproteinase-9 expression after hypoxia/reoxygenation [171]. In a rat model of traumatic brain injury, administration of calcitriol, the active metabolite of vitamin D, at 30 min, 24 h and 48 h after the insult, attenuated neurobehavioral deficits, brain edema and apoptosis as well as down-regulating NADPH oxidase, which may be protective against oxidative stress [172]. 

Although there is currently no evidence to suggest that vitamin D supplementation is neuroprotective in preterm infants, monitoring vitamin D status should be considered as a focus for future research [173]. Further animal studies investigating possible neuroprotective effects of vitamin D in a variety of preterm brain injury models would help to determine whether vitamin D may be a useful neuroprotective treatment for the preterm infant.

### 5.3. Cell-Based Therapies

There is increasing preclinical evidence that “stem” cell (i.e., pluripotent cells) therapy may be a viable neuroprotective strategy for the preterm brain, particularly after exposure to HI or infection/inflammation [174,175,176,177]. The potential neuroprotective efficacy of stem cell treatments appears to be mediated by multiple protective effects including promotion of proliferation, growth and differentiation through release of trophic factors and chemokines to support host cell survival and development, and their ability to modulate the inflammatory responses post-HI [175,176,177]. A variety of different stem cells are currently being assessed clinically and preclinically, including human amniotic epithelial cells (hAECs) [174,177,178] and umbilical cord blood (UCB). 

#### 5.3.1. Human Amnion Epithelial Cells (hAECs)

There are many different stem cells available. hAECs offer a number of practical advantages. They can be readily harvested from the amnion membranes, which surround the fetus in utero and are discarded at birth, therefore they do not require invasive extraction, making them readily available for rapid treatment in the early postnatal phase [155]. hAECs are pluripotent, are capable of differentiating into cell types of all three germ layers, are non-tumorigenic, non-immunogenic and have significant immunomodulatory properties, making them ideal as a generic therapy [175,179]. 

In adult mice, hAECs have been shown to reduce infarct development, reduce inflammation and functional deficits after stroke, while in the adult marmoset, hAECs also prevented infarct development [180]. Further, hAECS have been shown to reduce brain swelling and improve motor function after intracerebral hemorrhage and to reduce inflammation in experimental autoimmune encephalitis [181]. In a near-term fetal sheep model of infection/inflammation induced by lipopolysaccharide, hAECs administered at 0, 6 and 12 h after intra-amniotic administration of LPS, have been shown to ameliorate white and grey matter injury [182], in association with reduced microglial activation, suggesting that hAECs reduced inflammation. Similarly, hAECs reduced ventilation-induced inflammatory white matter injury in newborn lambs [183], as well as lung injury associated with ventilation [184], and hyperoxia [185]. 

hAECs also appear to be neuroprotective in the preterm brain after asphyxia. Delayed intranasal infusion of hAECs, given at 24 h and 3 and 10 days after 25 min of complete umbilical cord occlusion, was associated with improved brain weight, improved oligodendrocyte maturation and myelination and reduced microglia and astrocyte number after 21 days recovery in the preterm fetal sheep [186]. Further, hAECS have a relatively long therapeutic window of opportunity, showing similar anti-inflammatory effects when administered either 2 or 24 h after 25 min of complete umbilical cord occlusion in the preterm fetal sheep [187]. By contrast, hAECs did not reduce markers of neuroinflammation and injury in preterm lamb brains after mechanical ventilation; however, this was assessed at 48 h after birth and this may be too short a recovery period to see an effect [188].

#### 5.3.2. Umbilical Cord Blood (UCB) Cells

UCB contains a diverse mix of stem cells and progenitor cells, with the potential to generate a wide variety of cell types [189]. Advantages of UCB include that they are an abundant source of non-embryonic stem cells that are easily accessed in a non-invasive and risk-free manner and due to their immature nature, have remarkable proliferative potential [190]. UCB is a rich source of progenitor cells, regulatory T lymphocytes, mesenchymal stem cells, monocytes, endothelial progenitor cells and stromal precursor cells [191].

Early preclinical trials in neonatal rat pups showed that UCB administration at 24 h post-insult, decreased reactive gliosis and normalized connexin 43 expression, increased tissue repair and cognitive improvements and enhanced endogenous neural stem cell proliferation after HI induced by the Rice-Vannucci model of unilateral carotid ligation and inhalational hypoxia [175,192,193,194]. Further, in preterm fetal sheep, intravenous infusion of umbilical cord blood derived mesenchymal stem/stromal cells at 12 h but not 5 days after asphyxia induced by umbilical cord occlusion, reduced white matter injury and cerebral inflammation [195]. Further, UCB administered at 12 h was associated with a significant systemic increase in the anti-inflammatory cytokine Il-10 ten days after asphyxia as well as a reduction in oxidative stress [195]. When mesenchymal stem cells derived from umbilical cord blood were administered to preterm fetal sheep 12 h after asphyxia induced by 25 min of complete umbilical cord occlusion, preserved myelination, suppressed microglial activation, promotion of macrophage migration and accelerated self-repair within the preterm brain was seen [196].

Interestingly, the gestational age at which UCB cells are harvested may have an impact on the neuroprotective mechanisms. When preterm fetal sheep received UCB cells from either term or preterm sheep, both reduced white matter injury, cell death and microgliosis. However, only preterm cord blood prevented upregulation of plasma tumor necrosis factor alpha, while term cord blood increased the anti-inflammatory cytokine Il-10 and reduced oxidative stress [197].

## 6. Conclusions

Preterm birth is associated with a high burden of neurological impairment and lifelong disability. Developing therapies for brain injury in the preterm infant is particularly complex given the wide range of potentially injurious phenomena that preterm fetuses and infants may be exposed to before, during and after birth, including chronic antenatal and postnatal hypoxia, perinatal asphyxia, prenatal and postnatal infection/inflammation, poor respiratory function after birth, ventilation-induced brain injury and a variety of treatments with potential side effects, including anticonvulsants and antenatal and postnatal glucocorticoids. It is important to appreciate that each individual infant may be exposed to different combinations of these factors. The heterogeneous nature of preterm brain injury presents many challenges in the quest to develop treatment strategies for these infants. It is likely that different treatment strategies may be more effective in treating brain damage stemming from particular types of insults. For example, it is plausible that therapeutic hypothermia may alleviate brain damage after acute perinatal asphyxia but not chronic hypoxia. 

The multifactorial nature of preterm brain injury also presents a major challenge to the development of suitable preclinical models. Many preclinical studies, particularly those in large animal translational models focus on brain injury induced by acute asphyxia or infection/inflammation but studying the pathology associated with preterm birth itself or combinations of these factors is practically difficult in these paradigms. This has implications for the translation of potential new therapies into clinical trials where infants are likely to have been exposed to a variety of these potentially damaging factors.

Current treatments for preterm infants that may have beneficial effects on neurological outcome include antenatal glucocorticoids, which in addition to improving respiratory function, have been shown to reduce the incidence of IVH in preterm infants. The use of postnatal corticosteroids remains controversial due to concerns around increased risk of cerebral palsy but this appears to be influenced by dose and timing of administration. The use of anticonvulsants to treat seizures in the preterm brain remains controversial, due to poor efficacy and concerns around adverse effects in the developing brain raised by preclinical studies. Better understanding of the cellular mechanisms underlying seizure activity in the developing brain may aid in the development of safer and more effective anticonvulsant treatments for preterm neonates. There is conflicting evidence around the efficacy of MgSo4 for reducing preterm brain injury, with its use having been shown to be associated with a small but significant reduction in the risk of cerebral palsy and gross motor dysfunction in early childhood but no overall improvement in death or disability and studies with long-term follow up to school age to date have shown no long-term benefit.

A number of promising neuroprotective therapies are currently being investigated for use in the preterm brain. Preclinical studies suggest that therapeutic hypothermia is effective at reducing brain damage after asphyxia in the preterm brain but concerns remain around its safety. The results of the randomized controlled trial of therapeutic hypothermia in preterm infants are eagerly awaited to determine whether it is safe and effective for use in preterm infants who may have been exposed to a period of asphyxia around the time of birth. 

Further, preclinical studies suggest that recombinant erythropoietin may be a useful treatment option to reduce brain damage in the preterm infant but this has not yet translated successfully in clinical trials, perhaps due to the frequency of treatment. Preclinical studies suggest that melatonin, although the evidence for benefit is confound by the common use of ethanol as a diluent, human amnion epithelial cells and umbilical cord blood cells may also be viable treatment options for preventing brain damage in preterm infants. However, to ensure the greatest chance of these potential neuroprotective treatments translating into clinical practice, well-designed research in a range of translational models are essential to guide the development of evidence-based dosing regimens for use in clinical trials to ensure that they have the highest chance of being successful.

Despite the challenges associated with studying preterm brain injury and the development of treatment strategies, there are now a number of potential neuroprotective treatments showing promise in preclinical studies and/or in clinical trials. With further well-designed, translational research, these treatment strategies may help to reduce the high burden of brain damage and disability associated with preterm birth.

## Figures and Tables

**Table 1:** Summary of key clinical studies discussed in this review investigating the neurological effects of current and potential neuroprotective treatments in preterm infants.

Treatment	Study Details	Clinical Setting	Main Findings	Reference
Antenatal corticosteroids	Observational study 2323 infants (22–32 weeks) (EPIPAGE study)	Extreme or very preterm birth	Antenatal corticosteroids greatly increased survival with little evidence for effects on neurodevelopmental and behavioural outcomes at 5 years of age	[50]
	Meta-analysis of 30 trials 7774 women and 8158 infants	Preterm birth	Antenatal corticosteroids were associated with a reduction in perinatal death, neonatal death, respiratory distress syndrome, intraventricular haemorrhage, need for mechanical ventilation, and infection therapy	[51]
	Double-blind randomized controlled trial 1509 fetuses who received dexamethasone or betamethasone	Preterm birth	No significant difference in the incidence of survival without neurosensory disability at 2 years of age between dexamethasone and betamethasone	[54]
	Meta-analysis of forty-five trials (11,227 women, 11,878 infants) dexamethasone vs. betamethasone	Preterm birth	No difference between dexamethasone and betamethasone on neonatal death, neurodevelopmental disability, IVH and birthweight	[55]
Postnatal corticosteroids	18 premature infants (23–31 weeks) 7 treated with postnatal dexamethasone 11 not treated	Preterm infants with chronic lung disease	Postnatal dexamethasone was associated with impaired brain growth, particularly in cerebral cortical grey matter	[63]
	53 extremely low birthweight infants with high-quality MRI 11 infants received postnatal dexamethasone 30 infants received no treatment	Extremely low birthweight infants	Postnatal dexamethasone use was associated with smaller total and regional cerebral tissue volumes	[64]
	Cochrane Database systematic review of 32 randomised controlled trials including 4395 preterm infants with BPD who received early systemic corticosteroid treatment (< 8 days)	High-risk preterm infants	Early postnatal corticosteroids were associated with increased risk of abnormal findings on neurological examination and increased risk of cerebral palsy on long-term follow up	[65]
	Cochrane Database systematic review of 21 randomised controlled trials including 1424 preterm infants with BPD who received late systemic postnatal corticosteroids treatment (> 7 days)	Preterm infants with BPD	Postnatal steroids were associated with increased retinopathy of prematurity but not blindness, a trend towards a reduction in severe IVH and death but a trend towards increased cerebral palsy or abnormal neurological examination	[66]
	Meta-analysis of 16 randomised controlled trials comparing 1136 ventilated preterm infants > 7 days who received dexamethasone or placebo	Ventilated preterm infants	Higher cumulative doses of dexamethasone after the first week of life may decrease the risk of BPD without increasing the risk for neurodevelopmental impairment	[67]
Magnesium sulfate	Cochrane Database systematic review of 5 randomised controlled trials of antenatal magnesium sulfate therapy in women threatening preterm birth at less than 37 weeks gestational age including 6145 babies	Preterm birth	Antenatal magnesium sulfate therapy was associated with a small reduction in the incidence of cerebral palsy, with a number needed to treat of 63	[68]
	Systematic review of 6 trials involving Antenatal magnesium sulphate administration to women threatening preterm delivery before 34 weeks gestation including 5357 infants	Preterm birth	Antenatal magnesium sulphate therapy was associated with a significant reduction in the risk of cerebral palsy and substantial gross motor dysfunction	[69]
	Long-term follow up of a randomised controlled trial in women threatening preterm birth before 30 weeks gestation 535 received magnesium sulphate 527 received placebo	Preterm birth	Magnesium sulfate was not associated with improvements in neurological, cognitive, behavioral, growth, or functional outcomes in their children at school age (6–11 years)	[48]
	Long-term follow up of a randomised controlled trial of magnesium sulfate in women threatening preterm birth before 33 weeks gestation, including 503 children (7–14 years)	Preterm birth	Magnesium sulfate was not associated with any detrimental effects nor any significant effects on neurological outcome	[47]
Human recombinant Epo	Randomized control trial of rEpo versus placebo 800 infants (≤32 weeks)	Extreme or very preterm birth rhEPO at 500 U/kg i.v. every other day for 2 weeks starting within 72 h of birth	rhEpo decreased risk of death and moderate/severe disability at 18 month (13%) compared to control (26.9%)	[70]
	Randomized, double-blind trial 741 infants (24 weeks 0 days to 27 weeks 6 days)	Extremely preterm birth rEpo started <24 h of birth 1000 U/Kg every 48 h for 6 doses followed by 400 U/Kg 3 times/week	rhEpo did not reduce risk of severe developmental impairment at 2 years of age	[71]
	448 infants (26 weeks 0 days’ and 31 weeks 6 days)	Very preterm birth 3000 IU/kg iv within 3, at 12–18, and at 36–42 postnatal hours	rhEpo had no effect on neurodevelopmental outcomes at 2 years or 5 years	[72,73]
Therapeutic hypothermia	Retrospective study 31 preterm infants (34–35 weeks) 32 term infants	HIE	HT-associated complications in 90% of preterm infants and 81.3% term infants (*p* = 0.3) Preterms showed increased risk of hyperglycemia, early rewarming, death and white matter injury compared to term infants after cooling	[74]
	Retrospective uncontrolled cohort analysis 30 infants (33–35 weeks)	HIE	High incidence of complications, including coagulopathy (50%), thrombocytopenia (20%) and death (18.2%). Death or moderate to severe neurodevelopmental impairment occurred in 50% of infants with known outcomes	[75]
